# Supplementary figures and images for: Phenotypic, Physiological, and Gene Expression Analysis for Nitrogen and Phosphorus Use Efficienies in Three Popular Genotypes of Rice (Oryza sativa Indica)
Source: Plants (Basel). 2024 Sep 13;13(18):2567. doi: 10.3390/plants13182567 (PMC11434935; doi:10.3390/plants13182567)

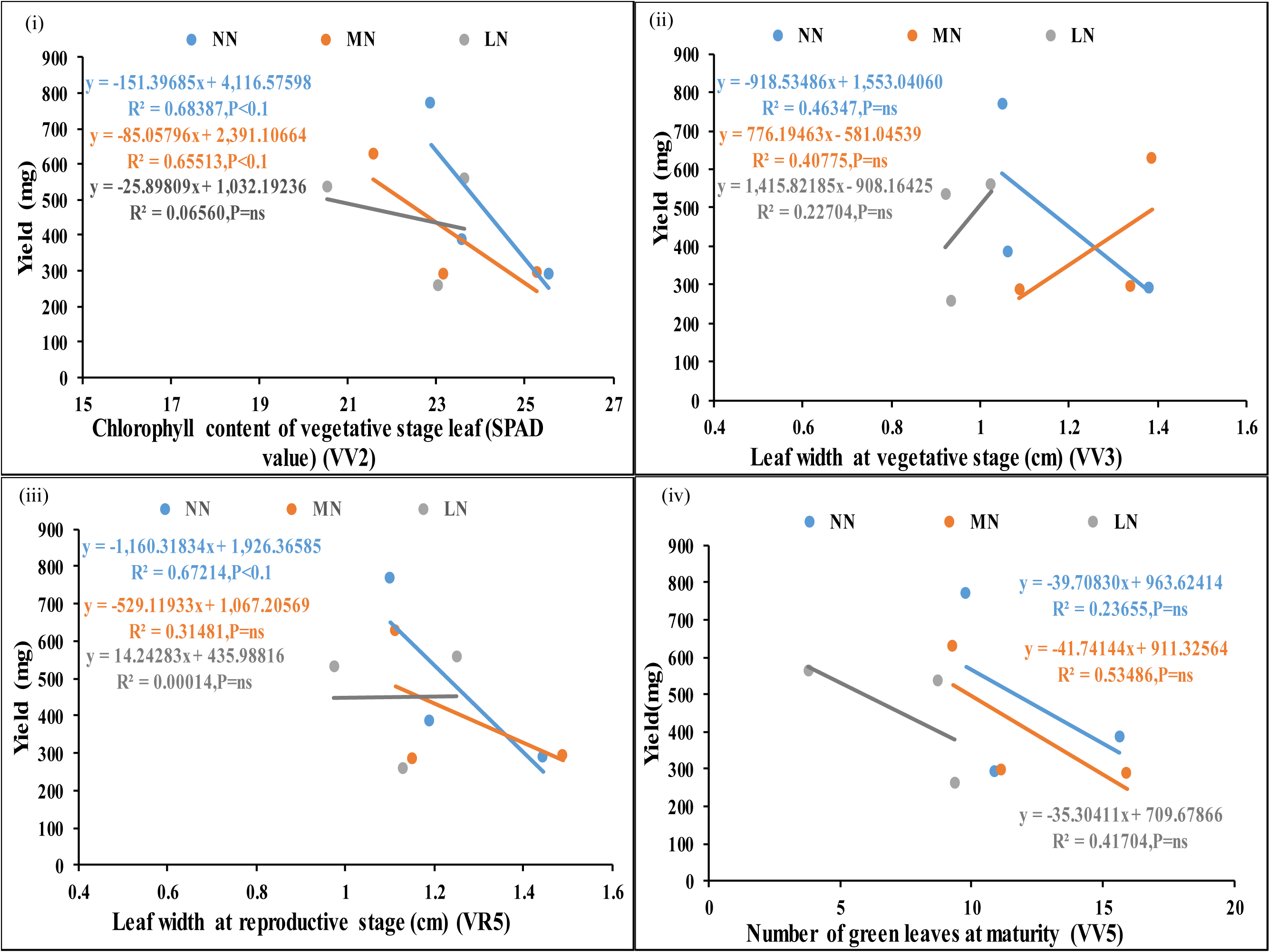

Supplement: Supplementary file 1 [file plants-13-02567-s001.zip › Supplementary Files MS/Supplementary figures/Supplementary Fig. 1.tif]

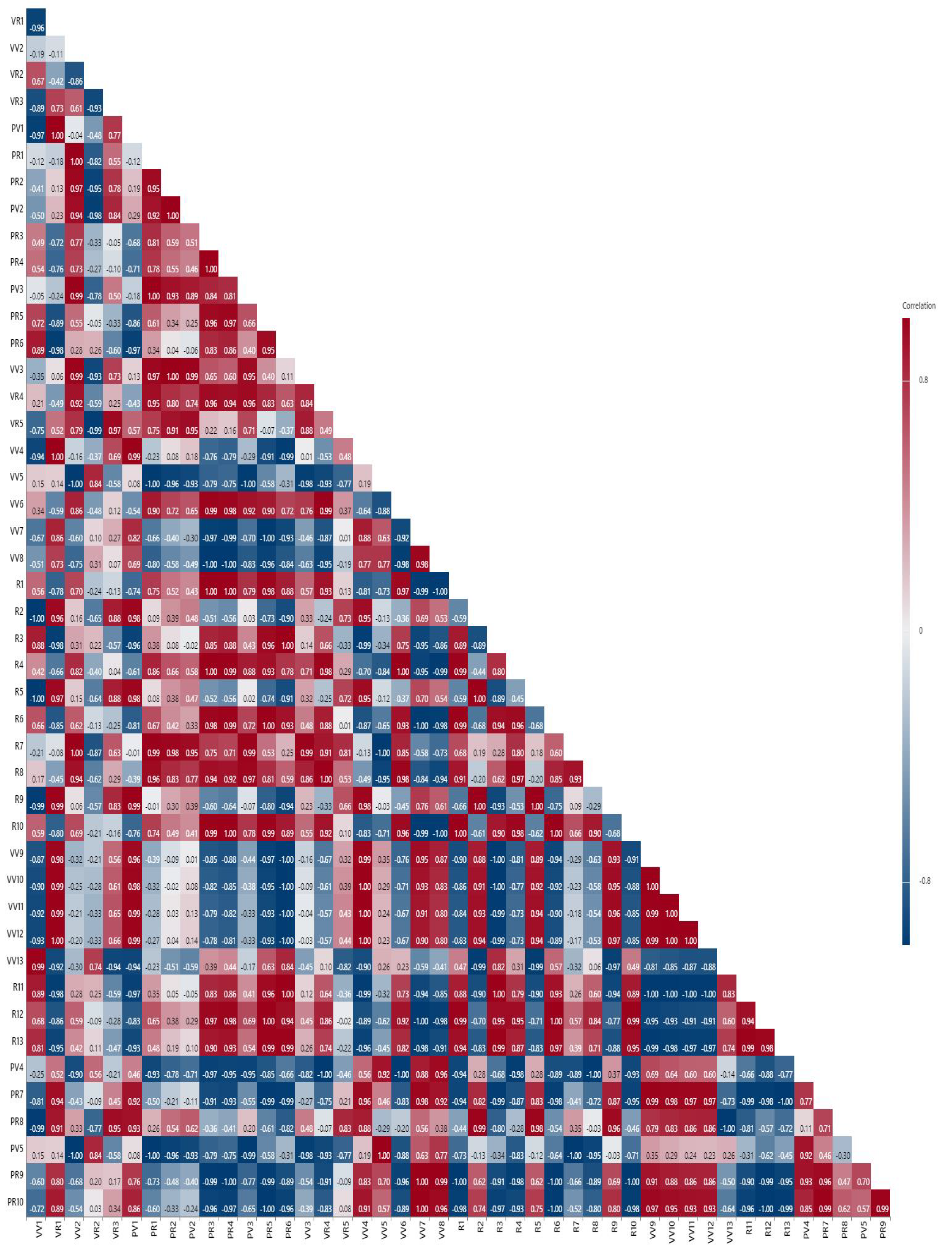

Supplement: Supplementary file 1 [file plants-13-02567-s001.zip › Supplementary Files MS/Supplementary figures/Supplementary Fig. 11(i).tif]

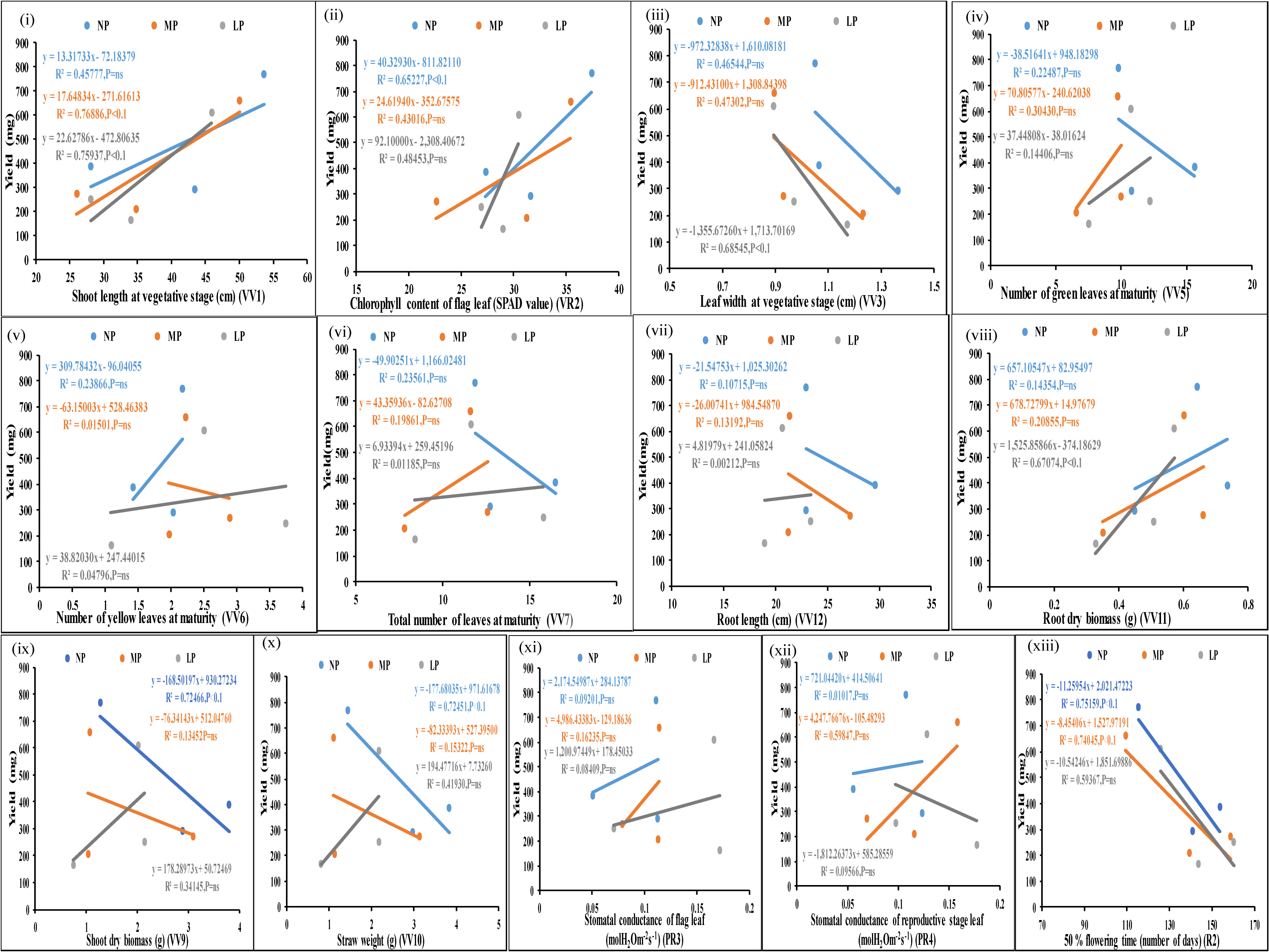

Supplement: Supplementary file 1 [file plants-13-02567-s001.zip › Supplementary Files MS/Supplementary figures/Supplementary Fig. 2.tif]

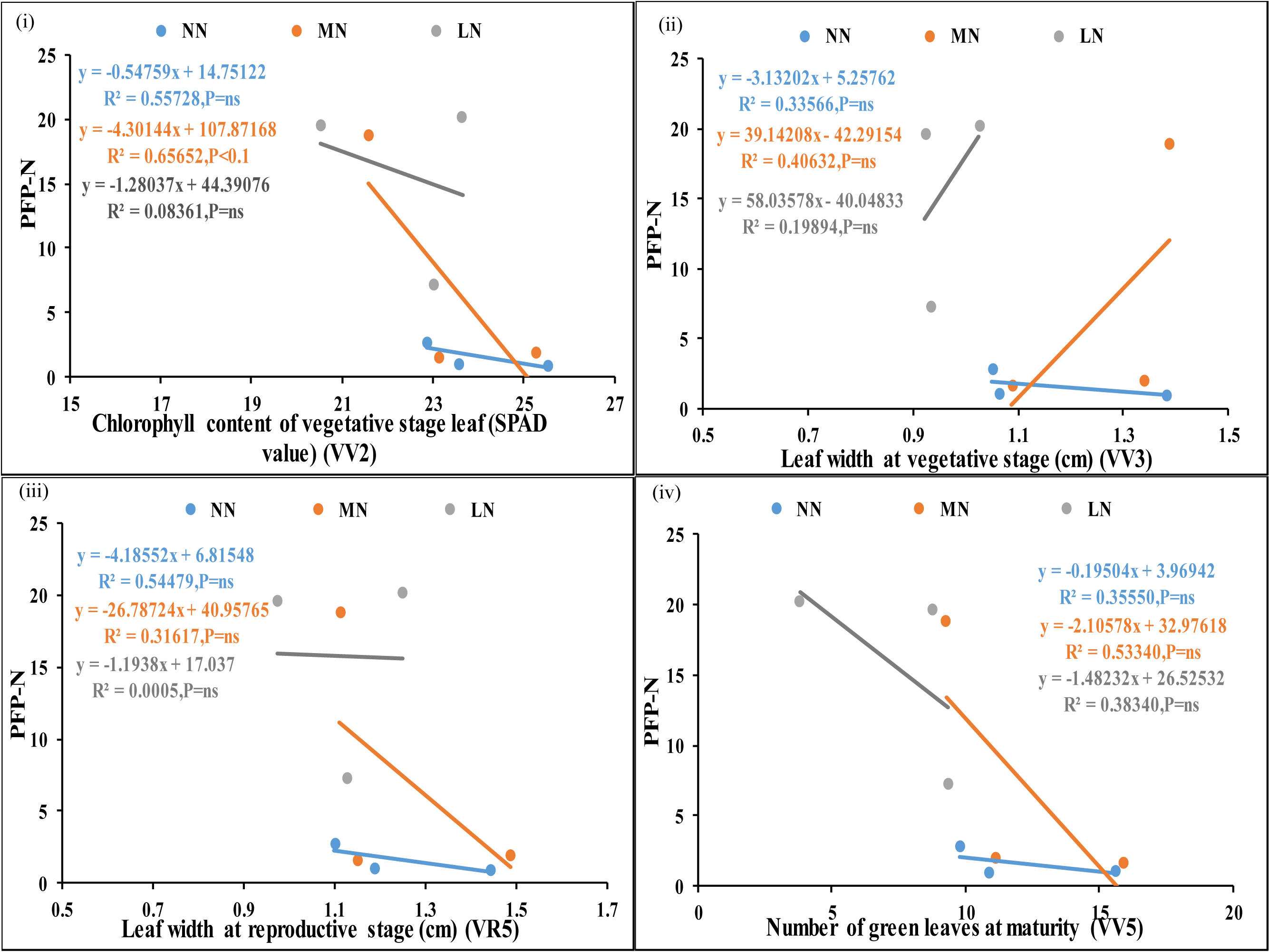

Supplement: Supplementary file 1 [file plants-13-02567-s001.zip › Supplementary Files MS/Supplementary figures/Supplementary Fig. 3.tif]

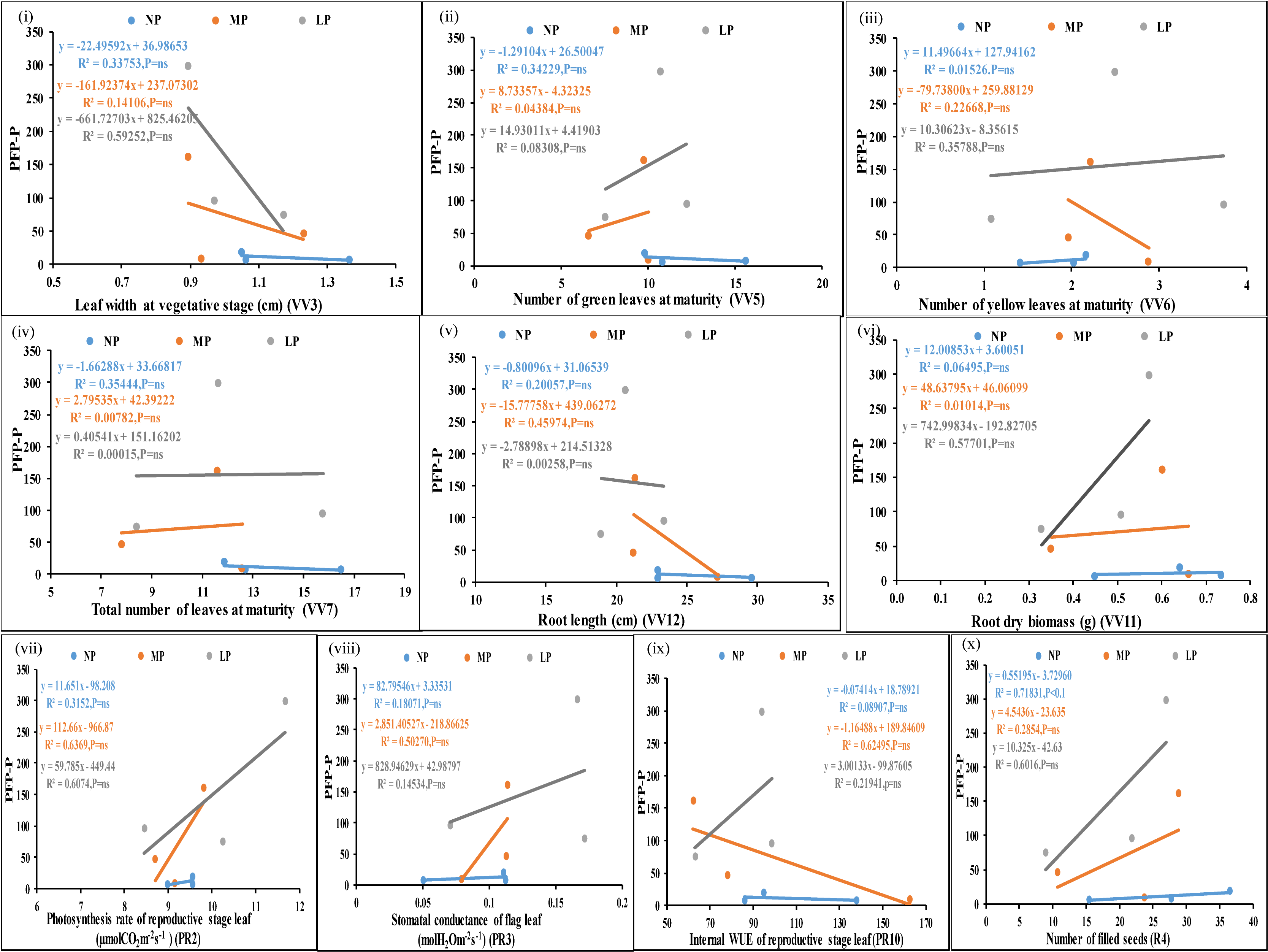

Supplement: Supplementary file 1 [file plants-13-02567-s001.zip › Supplementary Files MS/Supplementary figures/Supplementary Fig. 4.tif]

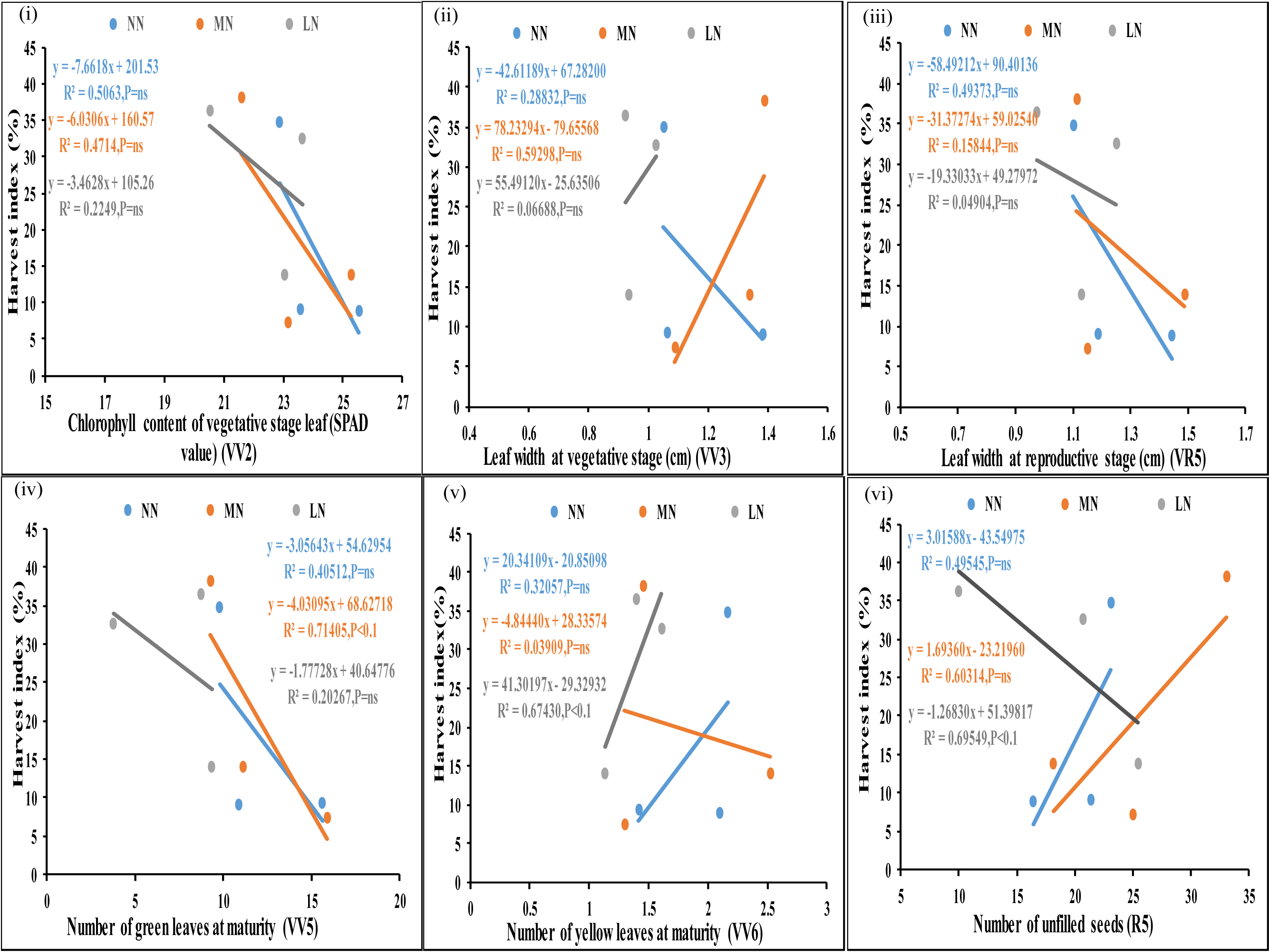

Supplement: Supplementary file 1 [file plants-13-02567-s001.zip › Supplementary Files MS/Supplementary figures/Supplementary Fig. 5.tif]

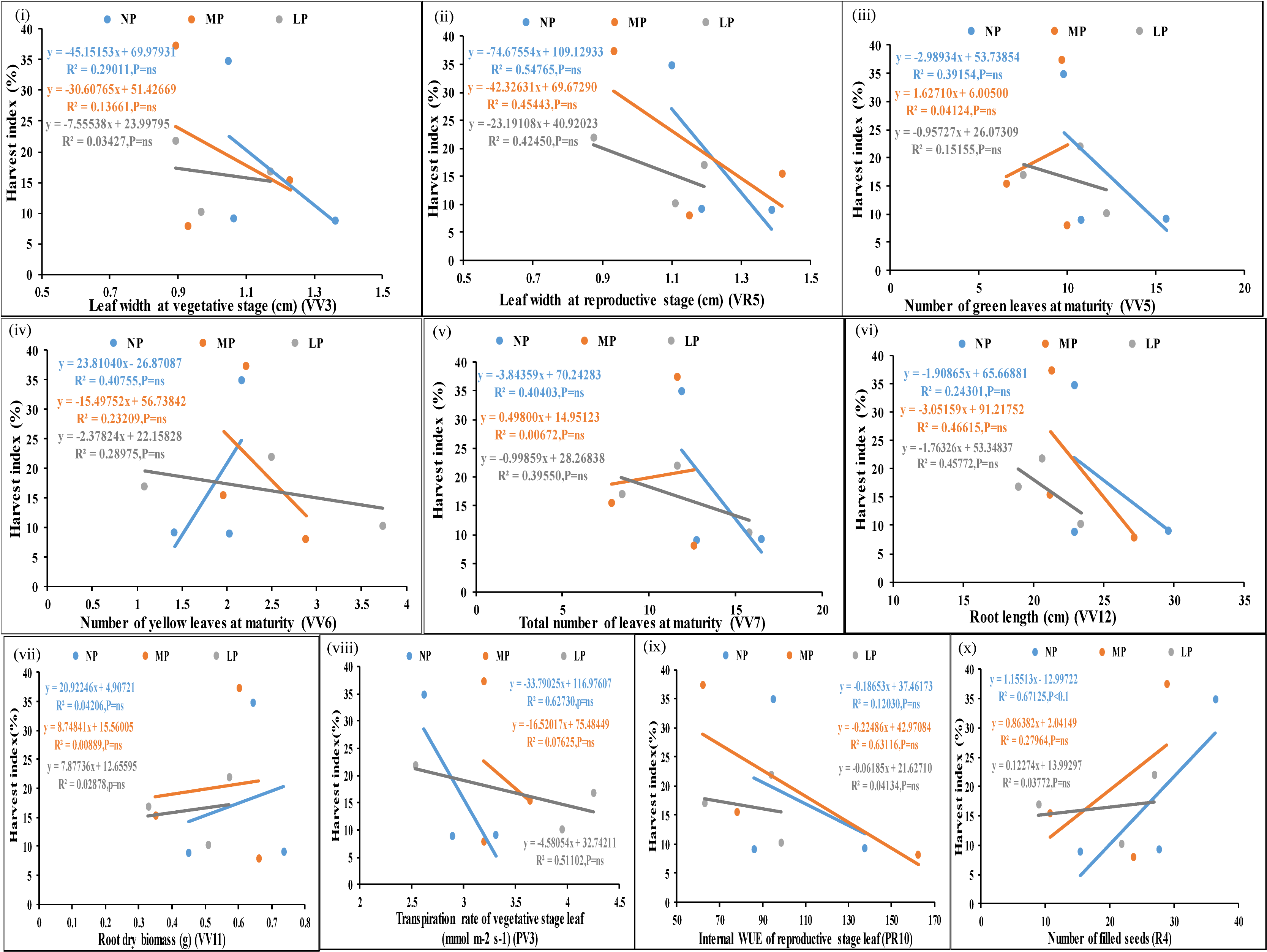

Supplement: Supplementary file 1 [file plants-13-02567-s001.zip › Supplementary Files MS/Supplementary figures/Supplementary Fig. 6.tif]

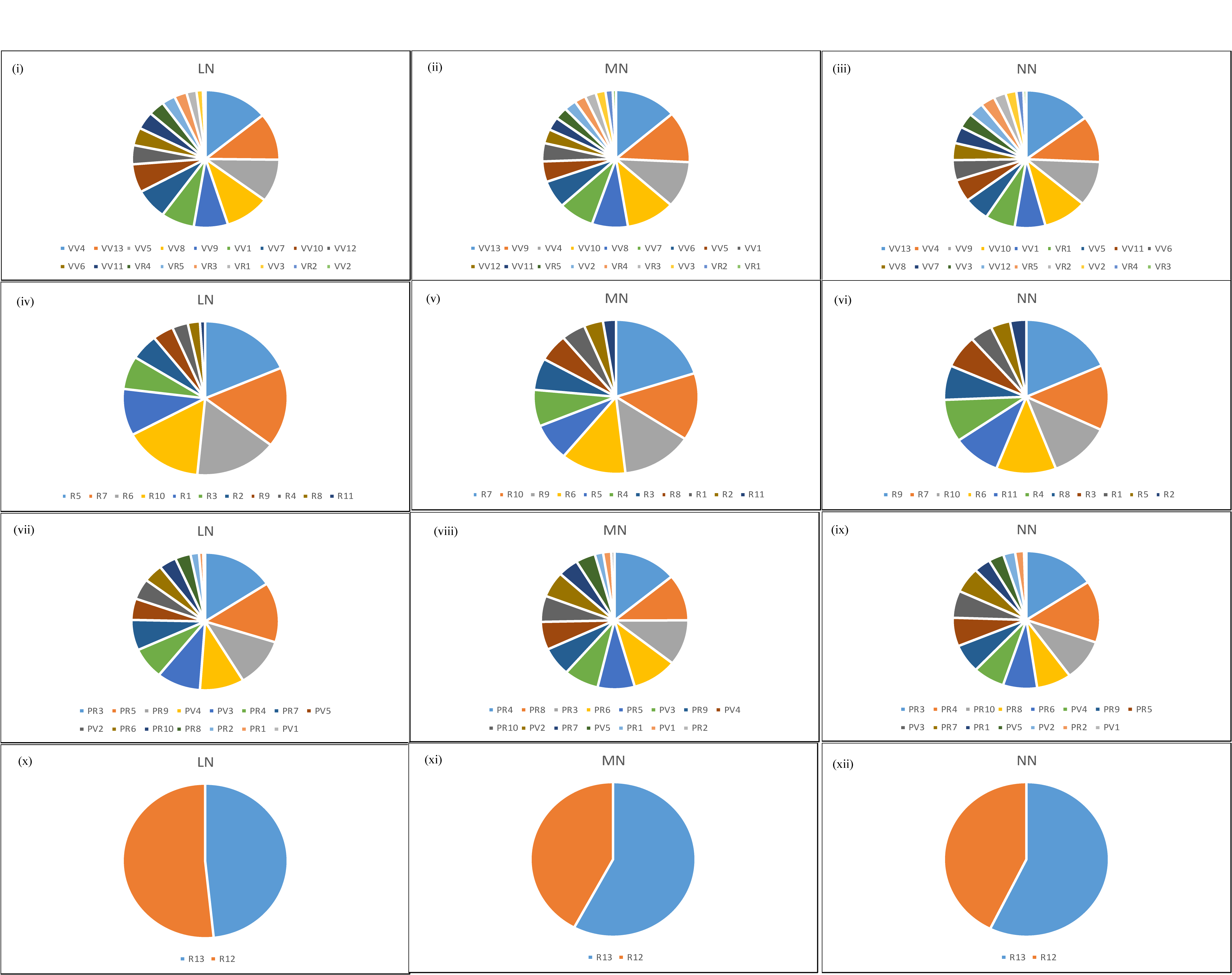

Supplement: Supplementary file 1 [file plants-13-02567-s001.zip › Supplementary Files MS/Supplementary figures/Supplementary Fig. 7.tif]

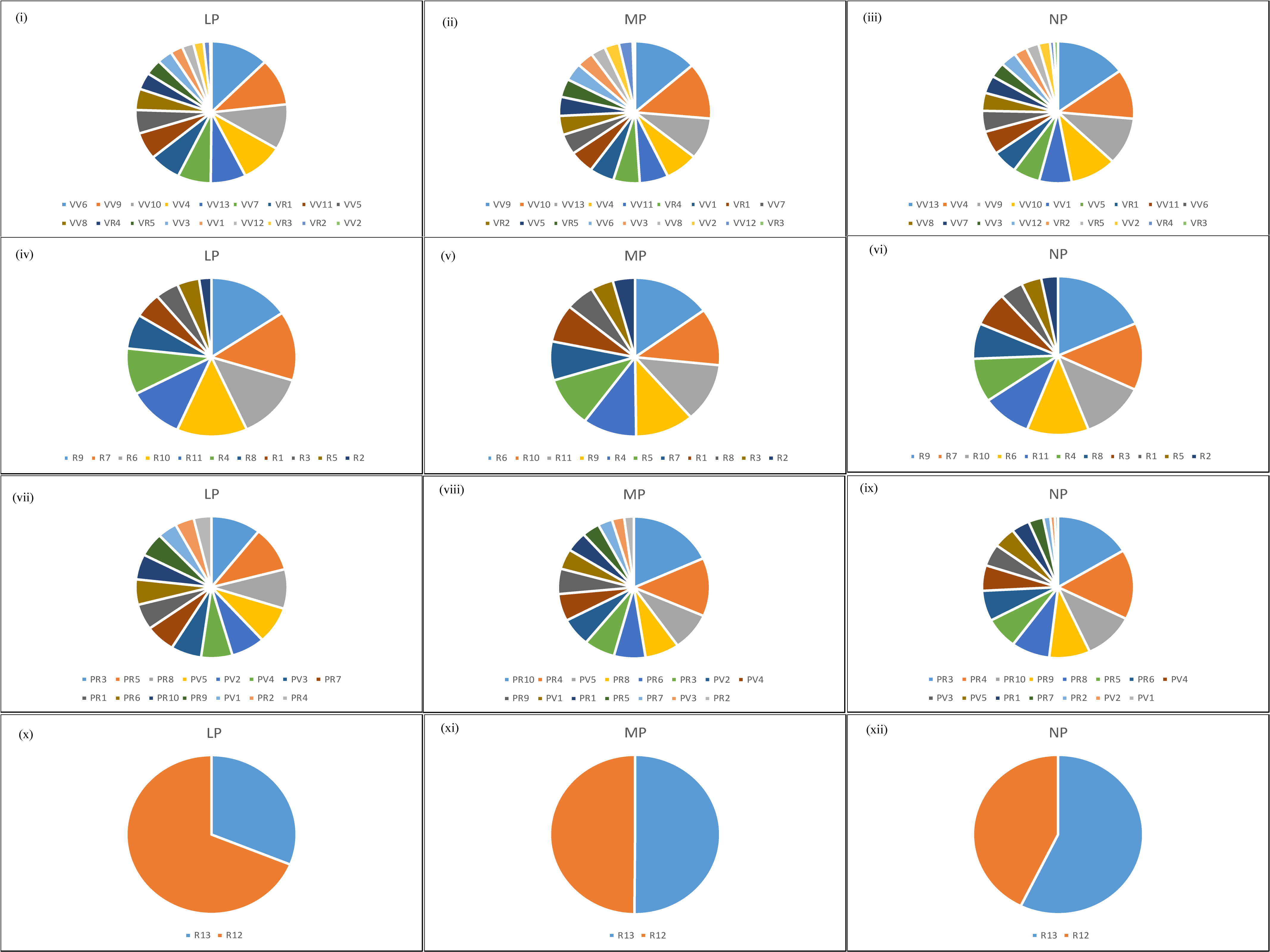

Supplement: Supplementary file 1 [file plants-13-02567-s001.zip › Supplementary Files MS/Supplementary figures/Supplementary Fig. 8.tif]

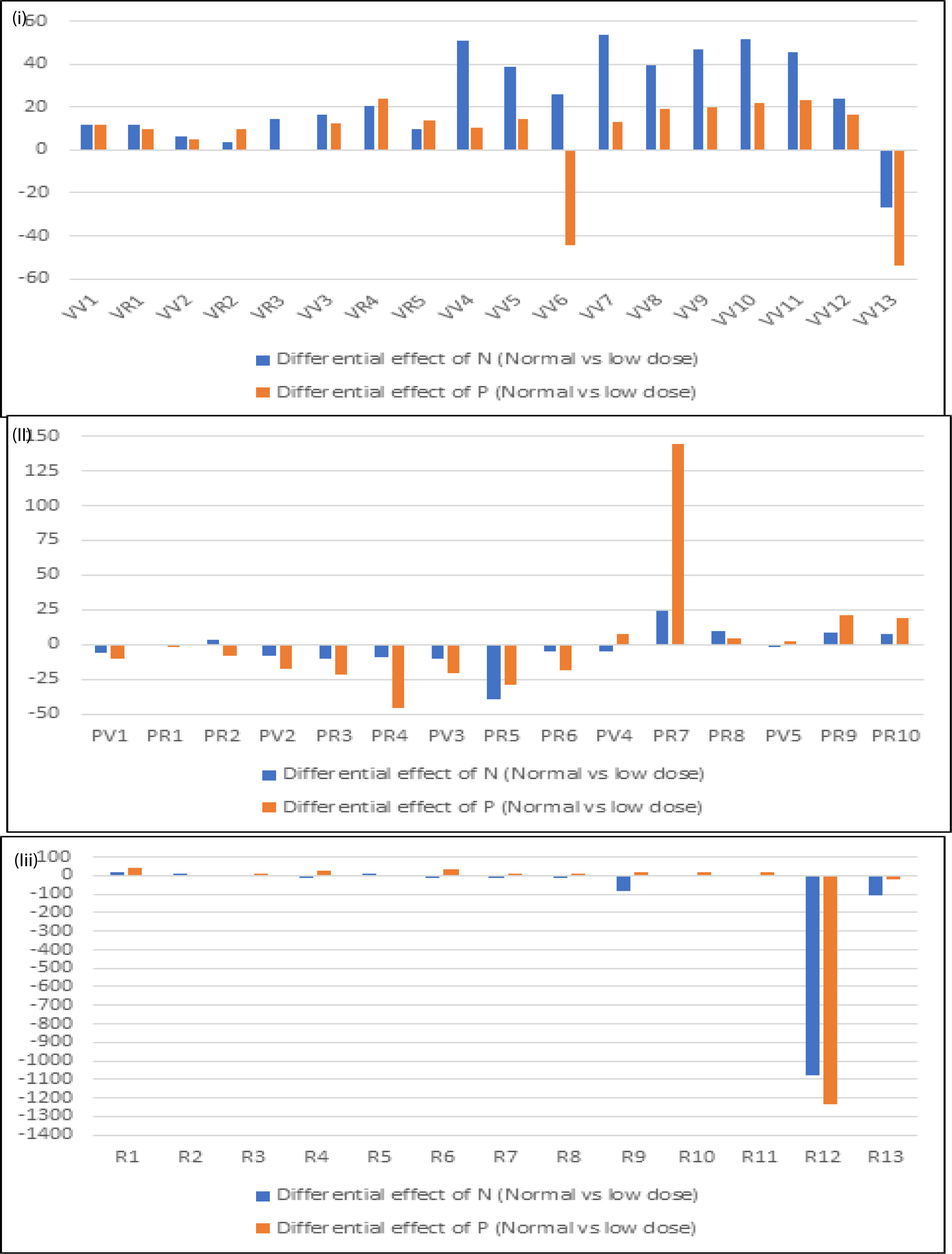

Supplement: Supplementary file 1 [file plants-13-02567-s001.zip › Supplementary Files MS/Supplementary figures/Supplementary Fig.10.tif]

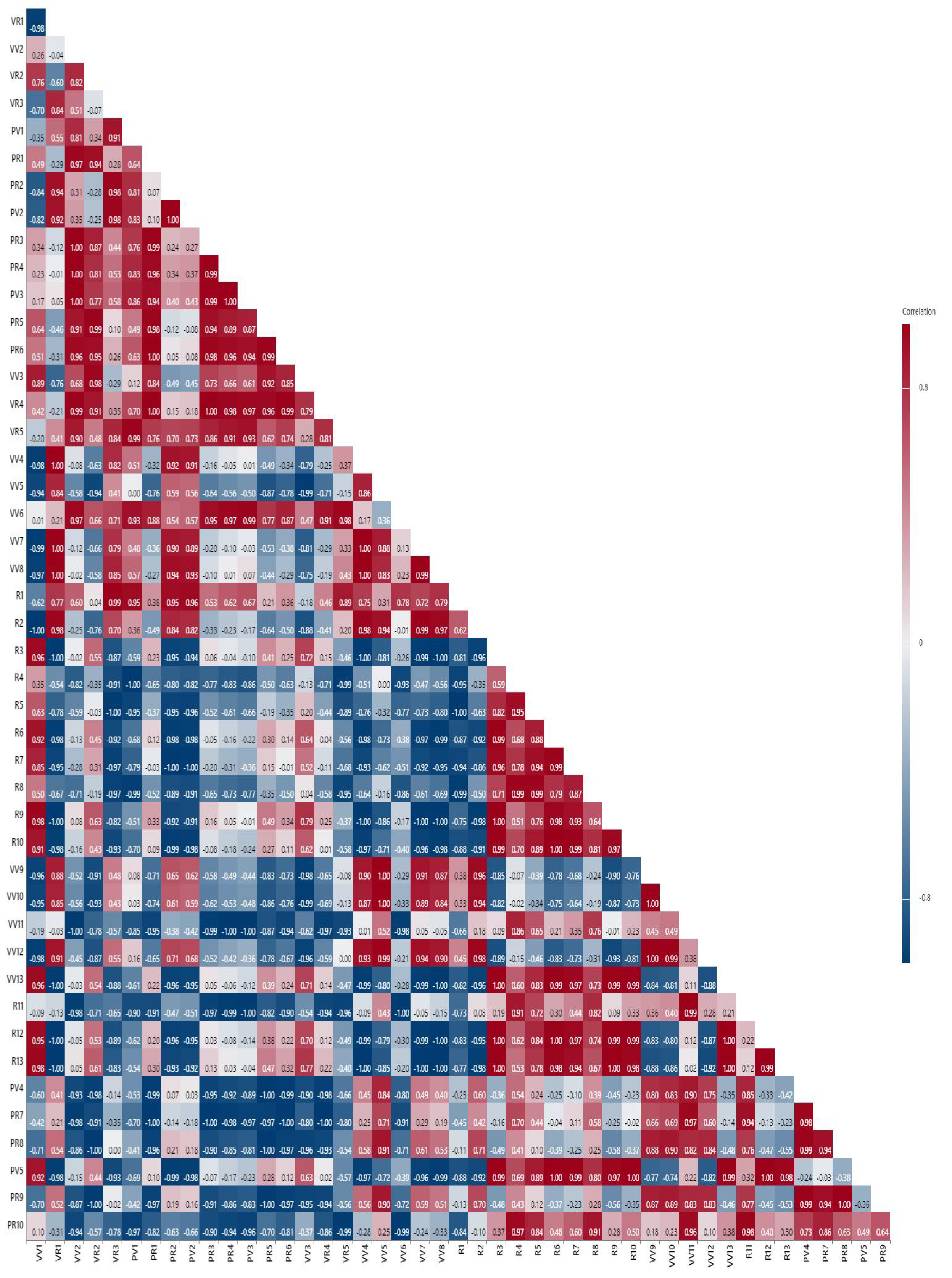

Supplement: Supplementary file 1 [file plants-13-02567-s001.zip › Supplementary Files MS/Supplementary figures/Supplementary Fig.11(ii).tif]

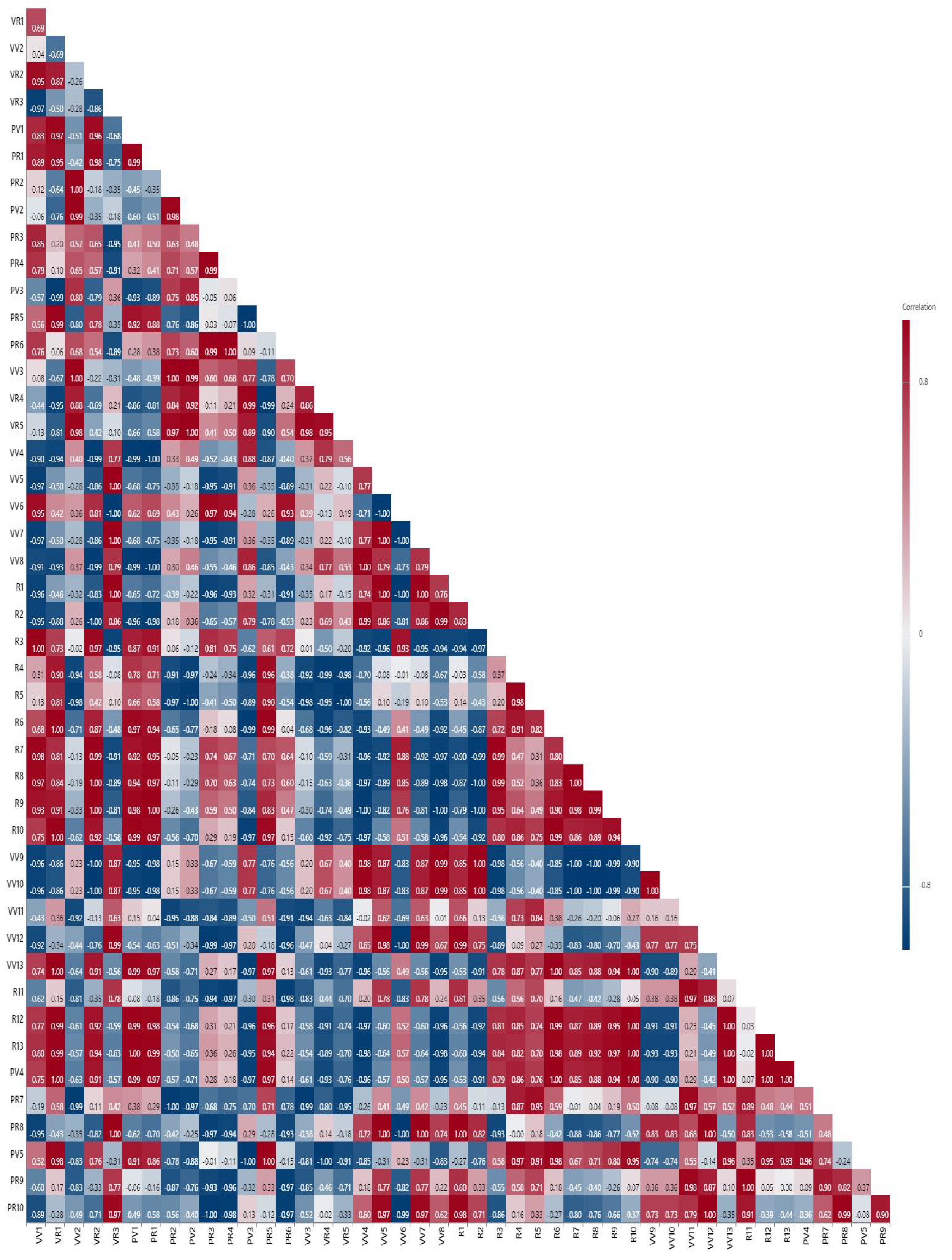

Supplement: Supplementary file 1 [file plants-13-02567-s001.zip › Supplementary Files MS/Supplementary figures/Supplementary Fig.11(iii).tif]

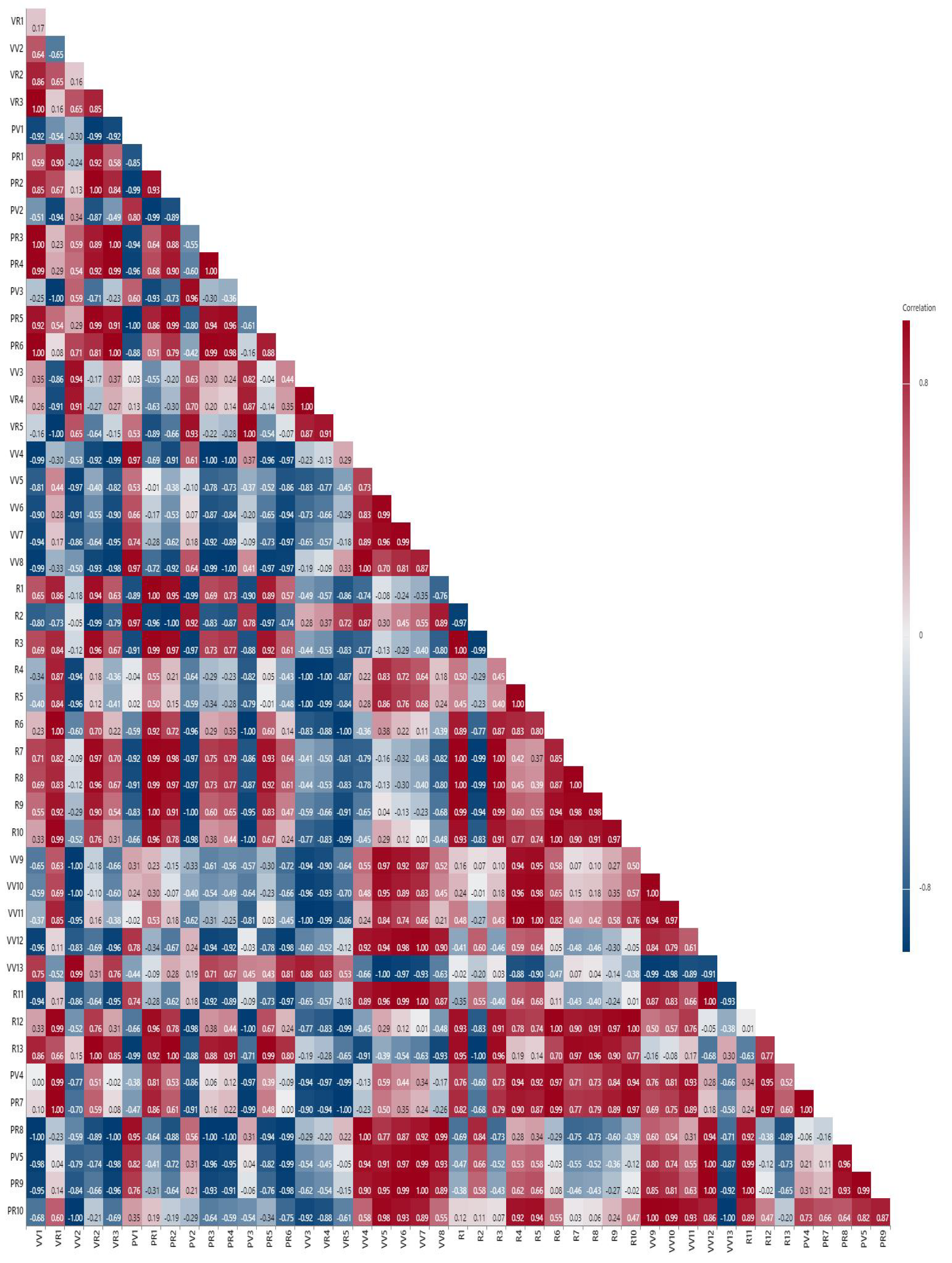

Supplement: Supplementary file 1 [file plants-13-02567-s001.zip › Supplementary Files MS/Supplementary figures/Supplementary Fig.12(i).tif]

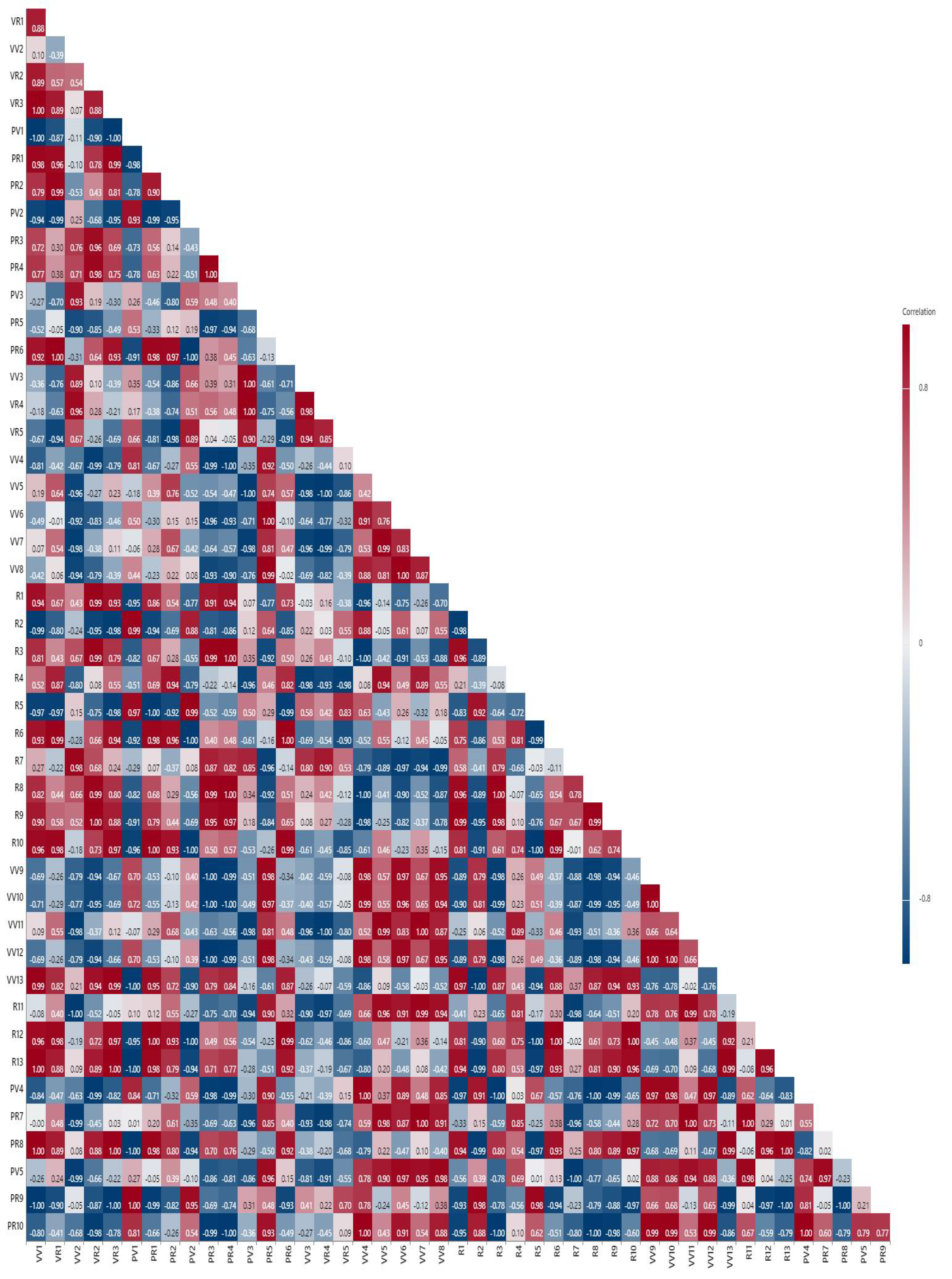

Supplement: Supplementary file 1 [file plants-13-02567-s001.zip › Supplementary Files MS/Supplementary figures/Supplementary Fig.12(ii).tif]

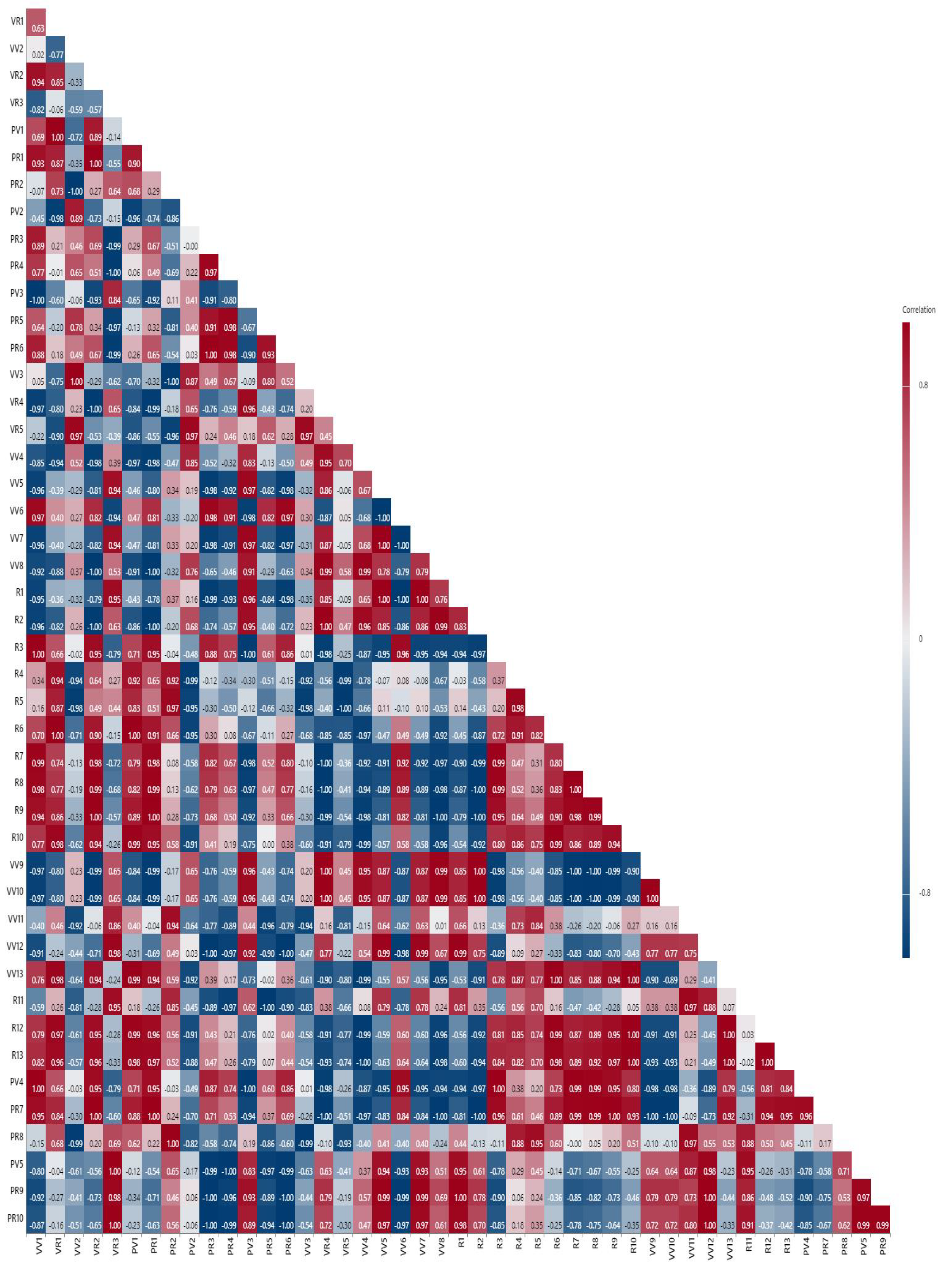

Supplement: Supplementary file 1 [file plants-13-02567-s001.zip › Supplementary Files MS/Supplementary figures/Supplementary Fig.12(iii).tif]

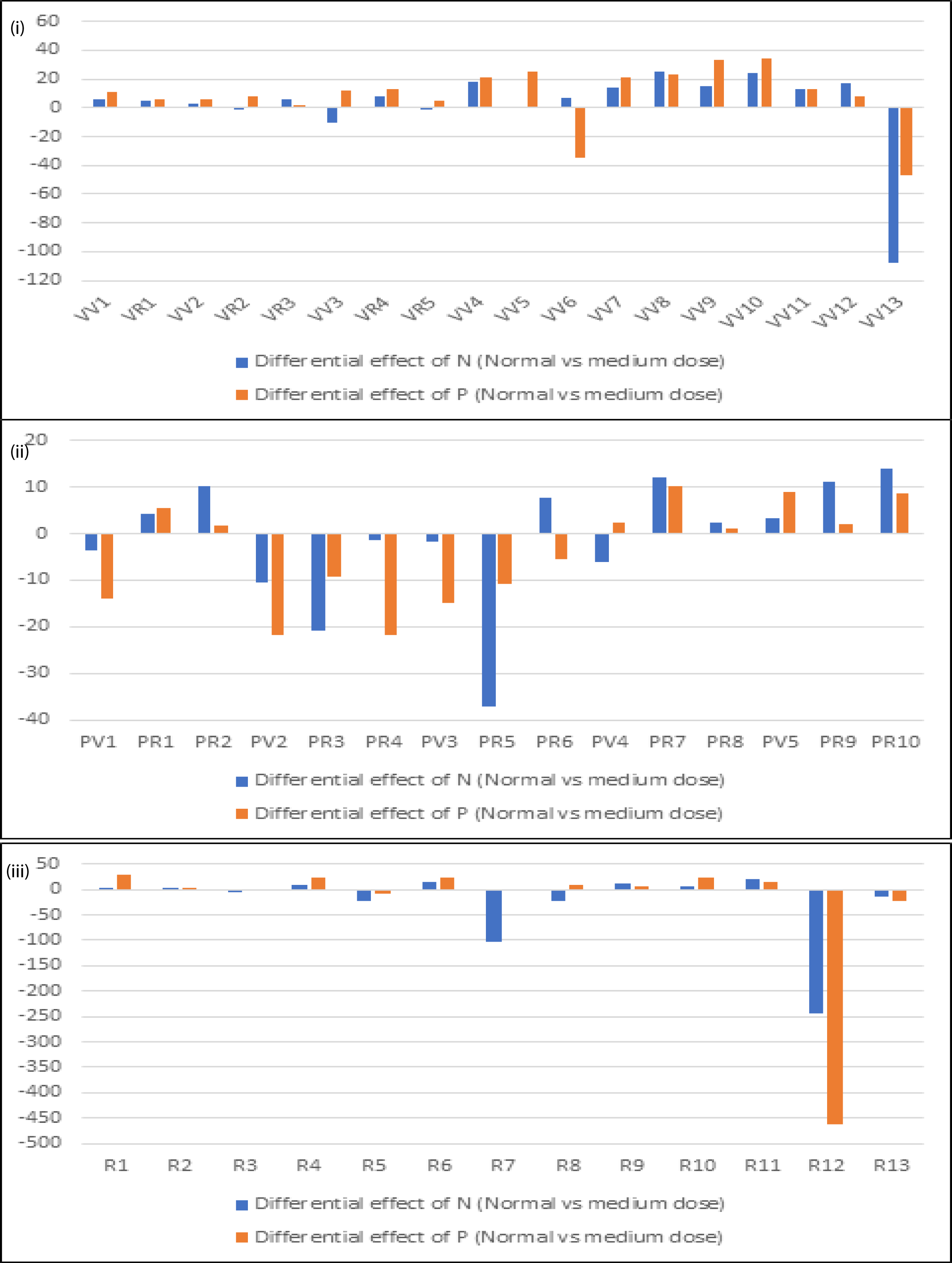

Supplement: Supplementary file 1 [file plants-13-02567-s001.zip › Supplementary Files MS/Supplementary figures/Supplementary Fig.9.tif]
